# FTH1 Pseudogenes in Cancer and Cell Metabolism

**DOI:** 10.3390/cells9122554

**Published:** 2020-11-28

**Authors:** Maddalena Di Sanzo, Barbara Quaresima, Flavia Biamonte, Camillo Palmieri, Maria Concetta Faniello

**Affiliations:** 1 Department of Experimental and Clinical Medicine, “Magna Graecia” University of Catanzaro, 88100 Catanzaro, Italy; adry@unicz.it (M.D.S.); quaresi@unicz.it (B.Q.); flavia.biamonte@unicz.it (F.B.); 2 Research Center of Biochemistry and Advanced Molecular Biology, Department of Experimental and Clinical Medicine, “Magna Graecia” University of Catanzaro, 88100 Catanzaro, Italy

**Keywords:** ferritin heavy chain (FTH1), pseudogenes, iron metabolism, miRNA, competing endogenous RNA (ceRNA)

## Abstract

Ferritin, the principal intracellular iron-storage protein localized in the cytoplasm, nucleus, and mitochondria, plays a major role in iron metabolism. The encoding ferritin genes are members of a multigene family that includes some pseudogenes. Even though pseudogenes have been initially considered as relics of ancient genes or junk DNA devoid of function, their role in controlling gene expression in normal and transformed cells has recently been re-evaluated. Numerous studies have revealed that some pseudogenes compete with their parental gene for binding to the microRNAs (miRNAs), while others generate small interference RNAs (siRNAs) to decrease functional gene expression, and still others encode functional mutated proteins. Consequently, pseudogenes can be considered as actual master regulators of numerous biological processes. Here, we provide a detailed classification and description of the structural features of the ferritin pseudogenes known to date and review the recent evidence on their mutual interrelation within the complex regulatory network of the ferritin gene family.

## 1. Introduction

Pseudogenes are genomic DNA sequences that are similar to functional genes but are defective due to the presence of premature stop codons, deletions/insertions, and frameshift mutations that abrogate their translation into functional proteins [1,2,3]. After their first characterization as non-functional residues of the genome’s evolution, it has been gradually discovered that an increasing number of pseudogenes play important biological roles, to the point that their initial remark as “junk DNA” today may be considered as being widely misunderstood [1,4]. However, the original sin of “Junk DNA” has deceptively guided the distinction between functional genes and non-functional pseudogenes in the process of annotating genomic regions that forms the basis on which an organism’s reference list of genes is built. As a consequence, the regions marked as pseudogenes have been and continue to be largely excluded from functional screens and genomic analyzes, perpetuating the delay in recognizing their biological role [1].

Based on the origin’s mechanism, pseudogenes are classified as (a) processed pseudogenes, which are derived from the retro-transposition of the processed mRNA; (b) unprocessed pseudogenes, resulting from the segmental duplication of an ancestor gene [1,5]. Together, these two categories of pseudogenes represent most of the entire genomic set of pseudogenes. Another less frequent type is (c) unitary pseudogenes, which are derived from an unduplicated ancestral gene that has undergone an inactivating mutation affecting its protein expression [1,6,7]. The processed pseudogenes represent the dominant type in mammals, whereas worm, fly, and zebrafish genomes are enriched for duplicated pseudogenes [8]. To date, about 20,000 pseudogenes have been identified in the human genome, and around 70% of these are unprocessed pseudogenes; moreover, 10% of human pseudogenes appear to be transcribed [1,9]. Evidence of pseudogene transcripts has further complicated the correct classification and understanding of the role of pseudogenes. On the one hand, evidence of transcription is useful for identifying coding genes that have been incorrectly annotated as pseudogenes; on the other hand, the experimental validation of the transcriptional status of pseudogenes is technically challenging.

Few examples of pseudogenes that have been reclassified as protein-coding genes demonstrate the importance of experimental validation of their functional activity [1]. The pseudogenes derived from retro-transcription (also known as retrocopies) have been classified as such based on some peculiar characteristics (absence of introns, a genomic polyadenylate tract, and target site duplications); however, some of them, such as Phosphoglycerate Kinase 2 (*PGK2)*, showed no gaps in the coding sequence and expressed a functional protein [1,7]. Similarly, the presence of interruption in the open reading frame of the parental gene is not a definitive criterion for pseudogene non-functionality, as demonstrated for the truncated form of Notch homolog 2 N-terminal-like (*NOTCH*2), Neurogenic Locus NOTCH Homolog Protein 2 (*NOTCH2NL*) [1,10,11]. In both cases, we are faced with pseudogenes that have been reclassified as protein-coding genes. At present, many intact retrocopies remain annotated as pseudogenes, and the experimental investigation could help to clarify their exact nature.

Besides these examples of pseudogenes that have actually turned out to be protein-coding genes, a wider series of transcriptionally expressed pseudogenes, whose actual translational status is unknown, have been described to perform biological functions through RNA-based regulatory mechanisms [1]. For example, some pseudogenes are transcribed as antisense RNAs with respect to the parental gene and can form a stable RNA duplex in vivo, inhibiting the translation of the parental gene [1]. Some other pseudogenes can be transformed into siRNAs [12] that regulate the parental gene, and others act as molecular sponges for miRNAs [13,14,15]. The general concepts related to the biological role of pseudogenes have been well described in some recent reviews, to which we refer the reader to for further information [1,2,9].

An analysis of pseudogenes distribution in the human genome has highlighted the fact that housekeeping gene families, such as ribosomal genes, tend to be enriched with a large number of processed pseudogenes, possibly due to the relatively constant expression level of housekeeping genes that increases their chances of being retrotranscribed [9,16]. The topic of this review is the human ferritin pseudogenes, which are part of a larger ferritin family which includes 4 genes and 47 annotated pseudogenes (NCBI Reference Sequence Database).

The best-known *FTH1* and Ferritin Light Chain (*FTL*) genes encode the two subunits forming the ubiquitous intracellular ferritin complex that performs the task of storing and releasing iron in a controlled manner [17]. The Mitochondrial Ferritin (FTMT) is a metal-binding protein located within the mitochondria and is endowed with a ferroxidase activity [18]. Lastly, ferritin heavy chain-like 17 (*FTHL17*) codifies a ferritin heavy chain-like protein that lacks ferroxidase activity [19]. *FTHL17* gene is mainly expressed in embryonic germ cells and has been associated with critical diseases, like cardiomyopathy, Familial Hypertrophic 9, and Friedreich Ataxia 2 [20]. All the ferritin family’s pseudogenes are annotated as processed pseudogenes, probably resulting from a retro-transpositional burst at the dawn of the primate lineage [16]. After underlining the salient features of the ferritin genes, this review will focus on the pseudogenes *FTH1*, whose biological function has been demonstrated or is plausible.

## 2. FTH1 and FTL

Ferritin is a 24-subunits spherical and hollow protein complex with an approximately 8 nm diameter cavity capable of storing up to 4500 iron atoms. The protein shell is highly conserved with a molar mass of around 450 kDa, and both the apoferritin and iron-loaded form have been well-characterized by a wide range of spectroscopic, crystallographic, and biochemical assays to determine their structure and function [21,22]. In eukaryotic cells, it is localized in the cytoplasm, nucleus [23], and mitochondria [18], thereby preserving iron in a non-toxic and readily available form. In addition to its intracellular form, ferritin is also an abundant protein in circulation. It is also present in plasma and its value can increase many times in different diseases, including cancer [24,25]. The 24 subunits of the cytoplasmic ferritin are made of two different types: the 19 kDa basic FLC subunit and the 21 kDa acid FTH1 subunit. FTH1 has ferroxidase activity, which specifically oxidizes ferrous iron (Fe^2+^) to ferric iron (Fe^3+^), while FTL is mainly associated with iron nucleation and stabilization of assembled ferritin proteins [26,27]. The ratio of these two chains is tissue-specific and controls iron storage and availability; the two subunits co-assemble into different types of isoferritins, which are either more acidic (H-rich) or more basic (L-rich), depending on the relative proportions of H and L chains, respectively. This ratio H/L, as well as total ferritin, is critical to cell survival. Indeed, FTL and FTH1 subunits are not interchangeable, and FTL is unable to compensate for the function of FTH1 in knockout mice [28,29].

In the nucleus, only the H-type subunit composes the ferritin shell. Although the nuclear ferritin functional role is still under investigation, a substantial body of evidence suggests that this protein may be involved in protecting DNA from oxidative damage and/or in the transcriptional regulation of a specific set of genes [30]. The role that nuclear FTH1 could play during neoplastic transformation is under discussion.

Various control mechanisms regulate the expression of *FTH1* and *FTL* coding genes [31,32,33,34,35], which affect the rate of transcription, the mRNAs stabilization and rate of translation, and the modulation of their half-life. The best-described mechanism so far is the translational control of the *FTH1* and *FTL* mRNAs, primarily in response to the intracellular iron content. Indeed, *FTH1* and *FTL* the translational efficiency of mRNAs is negatively regulated by iron regulatory proteins (IRP1 and IRP2), which bind to a specific domain, known as iron-responsive element (IRE), located in the 5’-UTR of the two mRNAs [36]. Since iron decreases the activity of IRP1 and destabilizes IRP2, the net effect of cellular iron overloading is an increase in iron storage via ferritin up-regulation. Differences in the post-transcriptional regulation of *FTH1* and *FTL* were not evaluated until 2007, when Sammarco et al. produced evidence that the two subunits responded independently to cellular iron concentrations, underscoring the importance of evaluating *FTH1* and *FTL* IREs separately [37].

Also, degradation of ferritin mRNA constitutes a mechanism by which iron can be released from ferritin and subsequently reutilized by cells. A different half-life of the two transcripts has been reported in different cell types, as well as a prolonged ferritin H mRNA half-life after ionomycin treatment [38].

Oxidized ferritin is recognized and degraded by the 20S proteasome, suggesting that this proteolytic system is involved in the turnover of abnormal ferritin [39]. Moreover, Nuclear Receptor Coactivator 4 (NCOA4) binds ferritin and targets it to autophagosome [40]. The autophagic degradation of ferritin, ferritinophagy, participates in the regulation of cellular iron homeostasis [41].

However, an analysis of ferritin’s structural/functional relationship based only on its central role in iron metabolism could be simplistic. Aside from its role as an iron-storage and antioxidant molecule, an emerging body of evidence identifies new ferritin roles in physiologic and pathologic processes. FTH1 regulates angiogenesis during inflammation and malignancy by binding to high molecular weight kininogen (HKa) and blocking its anti-angiogenic activity on endothelial cells in vitro and in vivo [42,43]. FTH1 may also physically interact with several signaling elements involved in critical cellular pathways, such as the C-X-C Motif Chemochine Receptor 4 (CXCR4) receptor, expressed in a variety of human malignancies and ALADIN protein (alacrima-acalasia-adrenal insufficiency neurological disorder), whose mutations determine the triple-A syndrome characterized by a deficiency of FTH1 nuclear import [44,45]; moreover, FTH1 binds, stabilizes, and activates p53 under oxidative stress [46]. FTH1 acts as a tumor suppressor in non-small cell lung cancer [47], breast cancer [48], and ovarian cancer [49] and as a tumor promoter in metastatic melanoma cells [50]. Moreover, *FTH1* knockdown determines an increased expression of a specific set of onco-miRNAs’ [51] activation of a *H19*/miR-675 axis [52] and severe protein misfolding in K562 erythroleukemia cells [53], as well as an increased chemoresistance in K562 and SKOV3 cells [54].

In the last decade, several groups have described miRNAs, a new class of modulators that repress the expression of target mRNAs, as an important player of ferritin regulation in normal and in neoplastic cells [55]. Finally, Jia Jia Chan et al. in 2018 identified the *FTH1* transcript and multiple *FTH1* pseudogenes as targets of several oncogenic miRNAs in prostate cancer [56].

## 3. *FTH1* Pseudogenes

To date, 24 sequences are annotated as *FTH1* pseudogenes on the NCBI Reference Sequence repository (October 2020). Three pseudogenes *FTH1P14*, *FTH1P19*, and *FTH1P27*, whose names might suggest they are *FTH1* pseudogenes, have been annotated as *FTHL17* pseudogenes (NCBI Reference Sequence repository). All of them exhibit the characteristics of the processed pseudogenes, derived from the retro-transposition of the *FTH1* processed mRNA. The evidence of transcript pseudogenes was experimentally confirmed for 11 of them (Table 1).

**Figure 1:**
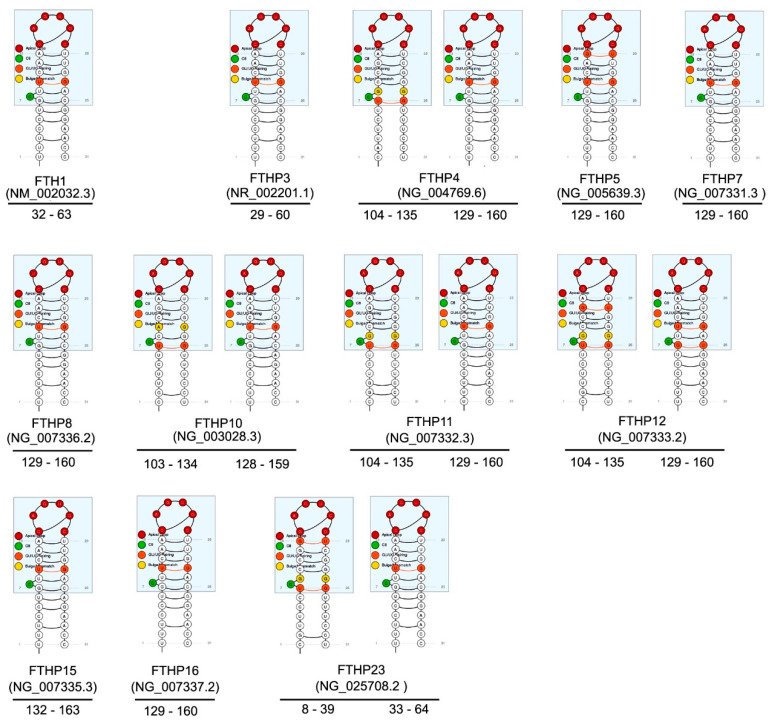
Predicted iron-responsive elements (IREs) in *FTH1* pseudogenes as retrieved by SIREs Server v2.0 [68]. For comparison, the upper left IREs structure refers to *FTH1* gene. The nucleotide positions of IREs in respect to the NCBI reference sequence are reported below each structure. Colored circles are as follows: red, Apical loop; green, Cytosine position 8 (C8); orange, GU/UG pairing; yellow, Bulge/Mismatch. Abbreviations used: FTH1P3, ferritin heavy chain 1 pseudogene 3; FTH1P4, ferritin heavy chain 1 pseudogene 4; FTH1P5, ferritin heavy chain 1 pseudogene 5; FTH1P7, ferritin heavy chain 1 pseudogene 7; FTH1P8, ferritin heavy chain 1 pseudogene 8; FTH1P10, ferritin heavy chain 1 pseudogene 10; FTH1P11, ferritin heavy chain 1 pseudogene 11; FTH1P12, ferritin heavy chain 1 pseudogene 12; FTH1P15, ferritin heavy chain 1 pseudogene 15; FTH1P16, ferritin heavy chain 1 pseudogene 16; FTH123, ferritin heavy chain 1 pseudogene 23.

An exciting aspect to be aware of is that some *FTH1*-pseudogenes contain one or more IRE sequences. For the expressed pseudogenes, these IRE sequences would potentially compete with *FTH1* for the IRE-binding factors. As we will see later, some transcript *FTH1*-pseudogenes compete with the *FTH1* gene for miRNA binding, contributing to the parental gene regulation. The competition of IRE sequences could constitute an additional level of the ferritin gene family’s complex regulatory network (Figure 1).

Some of the transcribed pseudogenes have the potential to produce homologous FTH1 polypeptides. Although to date, there is no experimental evidence of their existence, technical difficulties may have led to the lack of their recognition. Table 1 summarizes the main characteristics of the polypeptides that are possibly encoded by the transcribed pseudogenes of *FTH1*. It would be interesting, therefore, to verify whether they should continue to be considered as long non-coding RNAs (lncRNA), or they are actual protein-codifying genes erroneously annotated as pseudogenes.

*FTH1P3* is the most cited pseudogene in the literature, and we will discuss its importance in a separate following section.

*FTH1P1* was initially identified, along with *FTH1P2*, *FTH1P3*, *FTH1P4*, *FTH1P5*, *FTH1P7*, *FTH1P10*, *FTH1P11*, and *FTH1P12* following an effort to identify highly homologous sequences to FTH1 in hamster-human and mouse-human hybrid cell lines [57]. The presence of *FTH1P2* transcript has been determined in an experiment of RNA-pulldown as target of miR-638, along with *FTH1P8*, *FTH1P11*, and *FTH1P16* pseudogenes, although their NCBI Reference states that they have an “inferred” status [56,59]. *FTH1P16* on chromosome 11q13, corresponding to clone 133 reported by Costanzo et al., except for a G lacking at -54 in the 5’ noncoding region, was confirmed by Papadopoulos et al. and Quaresima et al. [59,63,64].

Circulating lncRNA *FTH1P2* (circRNA) was found enriched in plasma-derived exosomes of lung adenocarcinoma patients, along with *FTH1P11* [60], and were also found to be significantly related to the diagnosis and prognosis of non small cell lung cancer (NSCLC) [61]. The FTH1P2 ORF would potentially lead to a protein corresponding to the FTH1 carboxy region (aa 70–182) (Table 1). *FTH1P15* was identified in human-hamster hybrid cells during an effort to discover candidates for the hemochromatosis gene on the short arm of chromosome 6, a study that also confirmed the sequence of *FTH1P1* [58].

*FTH1P13* and *FTH1P14* were identified by restriction maps and southern analyses carried out by Zheng et al., who also confirmed the presence of all of the aforementioned pseudogenes [62]. *FTH1P6*, *FTH1P9*, *FTH1P24*, *FTH1P25*, and *FTH1P26* are a direct submission. *FTH1P22*, which was initially identified by Quaresima et al., is a pseudogene of *FTMT* (Gene ID: 100462772) [59]. *FTH1P21* and *FTH1P23* were mapped on chromosomes 4 and 3, respectively [65,66,67].

Recently, *FTH1P4*, *FTH1P10*, *FTH1P12*, *FTH1P16*, *FTH1P20*, and *FTH1P23* pseudogenes were validated in a microarray analysis of the differential expression of long-coding RNA in patients with the Epidermal Growth Factor Receptor (*EGFR*) wild-type group versus the *EGFR*-mutant group [61].

To determine the timing of the origins of *FTH1*- pseudogenes and *FTH1* parental gene, we performed a multiple sequence alignment with MegAlign Pro [69] and phylogenetic and molecular evolutionary analyses using MEGA version X [70].

**Figure 2:**
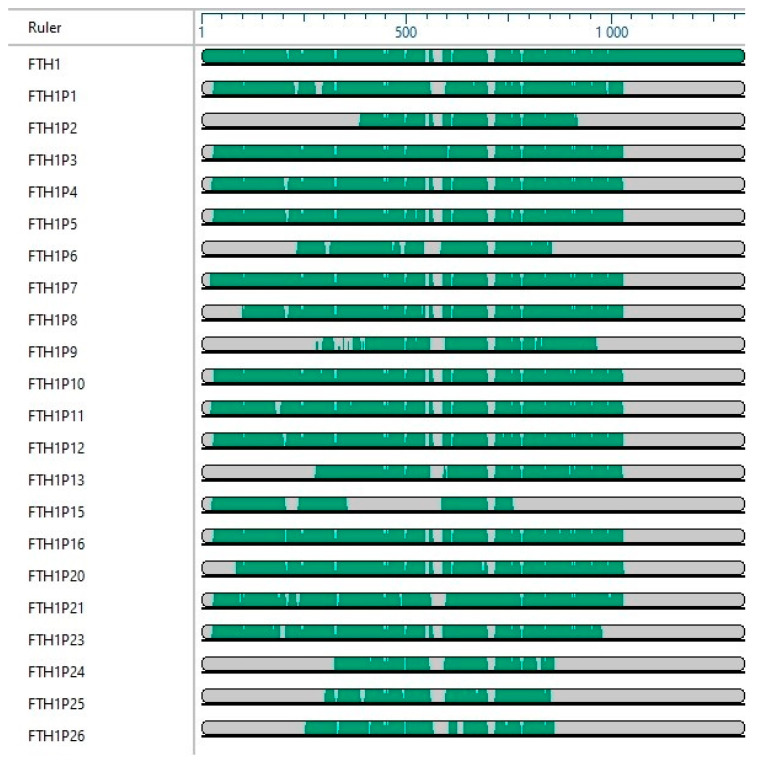
Multiple sequence alignment of the *FTH1* family using MegAlign Pro version DNA STAR, Inc.2020 [69]. In each “sequence block” the green areas representing regions of similarity between sequences while the gray areas representing gaps.

**Figure 3:**
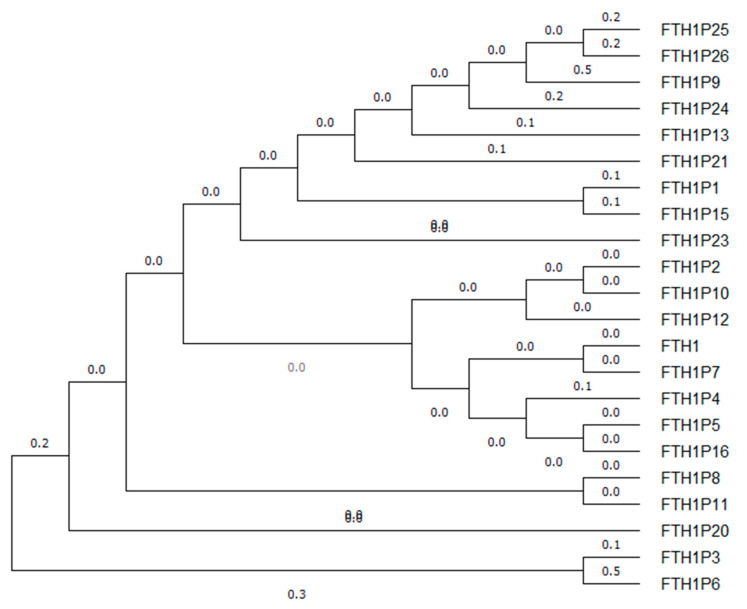
Evolutionary analysis of *FTH1* family by using the Maximum Likelihood method [70].

An overview of the degree of sequence identity of *FTH1* family and their evolutionary divergence is shown in Figure 2 and Figure 3.

The evolutionary history was inferred by using the Maximum Likelihood method and the Tamura-Nei model [71]. The tree with the highest log likelihood (−7970.55) is shown. Initial tree(s) for the heuristic search were obtained automatically by applying Neighbor-Join and BioNJ algorithms to a matrix of pairwise distances estimated using the Tamura-Nei model, and then selecting the topology with superior log likelihood value. The tree was drawn to scale, with branch lengths measured in the number of substitutions per site. This analysis involved 22 nucleotide sequences. There were a total of 1482 positions in the final dataset. Evolutionary analyses were conducted in MEGA X [70].

This analysis suggests that the pseudogenes *FTH1P6* and *FTH1P9* are the most evolutionarily distant pseudogenes from a common ancestor, followed by *FTH1P26*, *FTH1P25*, and *FTH1P24*, while *FTH1* and all the other pseudogenes are evolutionarily very close to each other and to the common ancestor.

## 4. Mitochondrial Ferritin (FTMT)

The mitochondrial ferritin is a homopolymer of the FTMT subunit encoded by an intronless sequence on chromosome 5q23.1, which shows characteristics of an *FTH1*-processed pseudogene: the absence of introns, a polyA tail residual, evidence of direct flanking repeats, and loss of the typical TATA or CCAAT box upstream of the ATG start codon [18]. Homologous *FTMT* genes have also been identified in plants, insects, and mammals. The FTMT subunit is characterized by more than a 75% sequence identity with H ferritin but differs from a more extended N-terminal targeting signal, which is cleaved after mitochondrial import occurs [18].

The protein is active in the mitochondrial matrix, where it can sequester and oxidizes iron due to the preservation of all the seven residues comprising the ferroxidase activity of FTH1 [72].

The transcriptional regulation of *FTMT* expression is accounted for by the −1884/−1 bp region upstream of the ATG codon, with the basal promoter activity located at 491 bp upstream of the transcription start site. The regulatory region contains both positive regulatory elements (−1128/−631) and negative regulatory elements (−631/−521). By using in silico analysis, DNA deletion analysis, and ChIP assay, six transcription factors were identified: YY1 (Yin Yang 1), CREB (c-AMP-response-element-binding protein), and SP1 (Specificity Protein 1), as activating elements; GATA binding protein 2 (GATA2), Forkhead-box protein A1 (FoxA1), and CCAAT-enhancer binding protein beta (C/EBP β) as inhibitor elements. The *FTMT* expression can also be regulated by a complex mechanism involving epigenetic events because of a densely methylated region upstream of the ATG codon [73].

FTMT is rich in tissues with high metabolic activity and oxygen consumption, suggesting that it could have a role in protecting mitochondria from iron-dependent oxidative damage. Its expression is not regulated by IRE/IRP, unlike the cytosolic ferritin, so its expression is not iron-dependent [74]. Despite the large amount of data on the functional role of FTMT, little is known about its expression regulation and the mechanisms of its specific cell/tissue expression.

FTMT is highly expressed in the ring sideroblasts of sideroblastic anemia patients, where it detoxifies the mitochondrial iron overload caused by the synthesis of the defective heme [75]. In brains that experience the symptoms of Alzheimer’s Disease, the FTMT overexpression induced by pro-inflammatory cytokines led to decreased amyloid-β production [76] while for the Restless Legs Syndrome, the increased FTMT expression contributes to the cytosolic iron deprivation [77]. Furthermore, high levels of FTMT was detected in cardiomyocytes of Friedreich Ataxia patients [20], while downregulation of FTMT was found in Neuroblastoma and Neurospongioma [78].

## 5. FTHL17

The X-linked *FTHL17* gene was originally discovered as a FTH1-like protein primarily expressed in spermatogonia [79,80]. While the human *FTHL17* seems to be a single gene located on Xp21.1 locus, the mouse *FTHL17* family includes six distinct genes on the XqA1 locus [19]. It was subsequently shown that FTHL17 has high levels of expression in tumors and low levels in normal somatic tissues; furthermore, the FTHL17 expression can be induced in cancerous cell lines by demethylating the DNA agents 5-aza-2′-deoxycytidine [81]. FTHL17 expression is downregulated during differentiation of mouse ESC into monolayers [19].

The *FTHL17* gene encodes a ferritin without ferroxidase activity for the substitution of the critical Glu62 into Lys that was shown to be sufficient to abolish this activity. Also, Glu61 is replaced with an Asp, while the other residues of the ferroxidase center are conserved. Another important feature was that FTHL17 is accumulated, at least in part, within the nuclei while FTH1 is mostly confined in the cytosol [19]. Since there is no signal of nuclear localization in FTHL17, the mechanism of its nuclear transport is unclear. Although its lack of ferroxidase activity suggests that FTHL17 does not have a role in protecting against oxidative damage and as iron storage, it seems to have regulatory functions, a hypothesis supported by its ability to localize in the nuclei and by its specific expression in germ cells [19]. Multiple pseudogenes of this gene have been found on chromosome X.

## 6. FTH1P3

*FTH1P3* is an intron-less pseudogene located in the 2p23.3 with a length of 954 nucleotides, which expresses a lncRNA in many tissues and cell lines [82]. *FTH1P3* is included in the lncRNA classification because of the presence of multiple early stop codons downstream of the AUG that prevent productive translation [82].

The promoter region of *FTH1P3* contains binding sites for the E2F transcription factor 1 (E2F1), which have been demonstrated to increase the transcription *FTH1P3* [83]. Moreover, *FTH1P3* contains iron-responsive elements (IREs) which act as post-transcriptional structured cis-acting RNA regulatory elements in the 5′ or 3′ untranslated regions (UTRs) of mRNAs [82].

LncRNA *FTH1P3* is up-regulated in several human cancers, including uveal melanoma [84], oral squamous cell carcinoma [85], esophageal squamous cell carcinoma [86], glioma [87], breast cancer [88], cervical cancer [89], and non-small cell lung carcinoma [90]. The increased expression of *FTH1P3* has been associated with proliferation, migration and invasion, promotion of epithelial-mesenchymal transition [90], and drug resistance [83]. Some mechanisms have been proposed to account for the oncogenic behavior of *FTH1P3*, whose most important mechanisms seems to be the involvement of FTH1P3 in some ceRNA pathways. This role of *FTH1P3* is discussed in a separate section of this review.

In NSCLC, *FTH1P3* RNA is mostly localized intranuclearly where it can bind the lysine-specific demethylase 1 (LSD1). By using co-immunoprecipitation experiments, Zheng et al. showed that the binding of *FTH1P3* to LSD1 would be important to recruit the demethylase activity of LSD1 on the promoter regions of the Metalloproteinase inhibitor 3 (*TIMP3*), thus epigenetically repressing *TIMP3* and accelerating the tumorigenesis of NSCLC. The E2F1/*FTH1P3*/TIMP3 axis seems to also be implicated in the gefitinib resistance of NSCLC [83].

Finally, Yang et al. have suggested a possible impact of FTH1P3 on the expression of SP1 and Nuclear Factor Kappa-light-chain-enhancer of activated B cell (NF-κB) (p65) in esophageal squamous cell carcinoma cells [86], although the evidence supporting this suggestion is still poorly consolidated.

In the last few years, *FTH1P3* has been identified in a group of up-regulated lncRNAs in oral squamous cell carcinoma (OSCC) and has been shown to function as an oncogenic regulator to facilitate OSCC progression by regulation of the miR-224-5p-Frizzled 5 pathway [85,91]. Moreover, it was shown that *FTH1P3* promotes the proliferation, migration, and invasion of oral squamous cell carcinoma by the association with Wnt/β-catenin via PI3K/Akt/GSK-3b signaling [92].

## 7. miRNA/FTH1 Family Network

Recent studies have shown that pseudogene-expressed RNAs can function either as antisense RNAs (also, endo-siRNAs) or as miRNA sponges [12,93,94]. The regulatory mechanism that involves miRNA sponges recall the concept of ceRNA, whereby pseudogene-expressed RNAs, messenger RNA, lncRNA, and circRNA may share the binding with the same miRNA through common miRNA response elements (MREs) [94,95]. The dysregulated expression of the former can alter the abundance of miRNAs, thus affecting the suppressive effect of miRNAs on the targeted transcripts. To be considered an optimal ceRNA network, the molecules involved in the ceRNA pathway would have to be in an almost equimolar ratio, and this criterion may discriminate between an effective and a less probable ceRNA pathway [96,97]. The involvement of pseudogene-derived transcripts in ceRNA mechanism has been demonstrated in many studies; here we will focus on ferritin pseudogenes and refer you to other reviews for a more general view of gene/pseudogene networks.

The *FTH1P3* is the pseudogene most cited in the literature for its involvement in the ceRNA networks. The role of lncRNA *FTH1P3* in ceRNA networks has been reported in studies that investigated the evidence of increased *FTH1P3* expression in some tumors, supporting the oncogenic role of *FTH1P3*. LncRNA *FTH1P3* contributes to the progression of OSCC, uveal melanoma, and glioma by functioning as a miR-224-5p sponge [84,85,87]. The mature miRNA microRNA-224-5p (miR-224-5p, previously named miR-224) is involved in a series of biological processes, including cell proliferation, migration, and invasion, in various malignancies [98,99,100,101,102]. Putative miR-224-5p binding sites on *FTH1P3* map at the 619–640 and 820–831 regions of the 954 lncRNA nucleotides [85]. In uveal melanoma and OSCC, the overexpression of *FTH1P3* decreased miR-224-5p expression and promoted the expression of Frizzled 5, which was the direct target genes of miR-224-5p; in uveal melanoma, the expression of Ras-Related C3 Botulinum Toxin Substate 1 (Rac1) is also affected by the influence of *FTH1P3*/miR-224-5p [84,85].

The role of lncRNA *FTH1P3* in ceRNA networks has also been discussed by Wang et al., which confirmed the potential regulatory mechanism of *FTH1P3* on breast cancer paclitaxel resistance through the miR-206/ABCB1 axis, thus providing a novel insight for the breast cancer chemoresistance [88].

Many miRNAs are able to target *FTH1*, including miR-200b, miR-181a-5p, miR-19b-1-5p, miR-19b-3p, miR-210-3p, miR-362-5p, miR-616-3p, and miR-638. [55,56].

Since pseudogenes have high sequence homology with parental genes, they may also share a pool of miRNAs that bind to common MREs, and hence serve as miRNA sponges to regulate parental RNAs. The lncRNAs of the pseudogenes are therefore endowed with a competitive ability against the parental gene, which is based on the sponging of common miRNAs. This competitive ability is theoretically more relevant for gene families made up of a large number of pseudogenes, and the ferritin gene family belongs to this group. Indeed, the *FTH1* gene:pseudogenes network has been reported to be relevant in prostate cancer, possibly by affecting iron balance [56]. By miRNA pulldown experiments, *FTH1* and some of its pseudogenes (*FTH1P2*, *FTH1P8*, *FTH1P11*, and *FTH1P16*) were identified as targets for miR-181a-5p, miR-19b-1-5p, miR-19b-3p, miR-210-3p, miR-362-5p, miR-616-3p, and miR-638, which also showed ceRNA activity [56]. In DU145 and PC3 models of prostate cancer *FTH1* and *FTH1P11* and *FTH1P16* show near equimolar expression level, further underlining the fulfilment of an important criterion for an effective parental gene:pseudogene ceRNA network [56]. As described in the previous section, *FTH1* expression is regulated by iron availability, whereby an iron excess induces *FTH1* expression and, conversely, iron depletion provokes *FTH1* downregulation. Thus, providing or removing iron to cells is a physiological approach to fine regulate FTH1 expression.

Interestingly, in DU145 and PC3 models of prostate cancer, the addition of iron induced the expression not only of *FTH1*, but also of *FTH1P11* and *FTH1P16*, two pseudogenes whose expression is not regulated by IREs, while iron depletion down-regulated *FTH1*, *FTH1P11*, and *FTH1P16* [56]. This type of experimental demonstration, whereby physiologically modulating the expression level of a gene of the ceRNA pathway, a modification in the same direction of the corresponding gene of the same pathway is observed provides more robust and convincing evidence of the efficacy of the ceRNA pathway, especially when compared with the most common approaches of ectopic overexpression or downregulation of gene/pseudogenes. Further investigation with mutated MREs, confirmed that the reciprocal regulation of *FTH1* and *FTH1P11/16* expression was mediated by ceRNA interactions [56].

## 8. Conclusions

Since the link between iron balance, oxidative stress, and tumorigenesis is widely recognized, the parental *FTH1*/pseudogenes ceRNAs could have a much broader role in oncology than that shown so far. Indeed, the ceRNAs identified in the prostate cancer model may also have an impact in other tumor settings; likewise, other lncRNA of ferritin pseudogenes may act in a similar way, which is why the measurement of lncRNA-*FTH1*-pseudogenes expression levels is desirable in those tumor models in which *FTH1*, iron balance, and oxidative stress appear to be involved. This approach would provide an additional level of understanding of the intricate connections between the various levels of gene expression and biological outcomes of cancer relevance, such as proliferation, survival, metastasis, and drug resistance, and would represent a paradigmatic example to extend to those oncogenes or tumor suppressor genes represented by a large family of pseudogenes.

**Figure 4:**
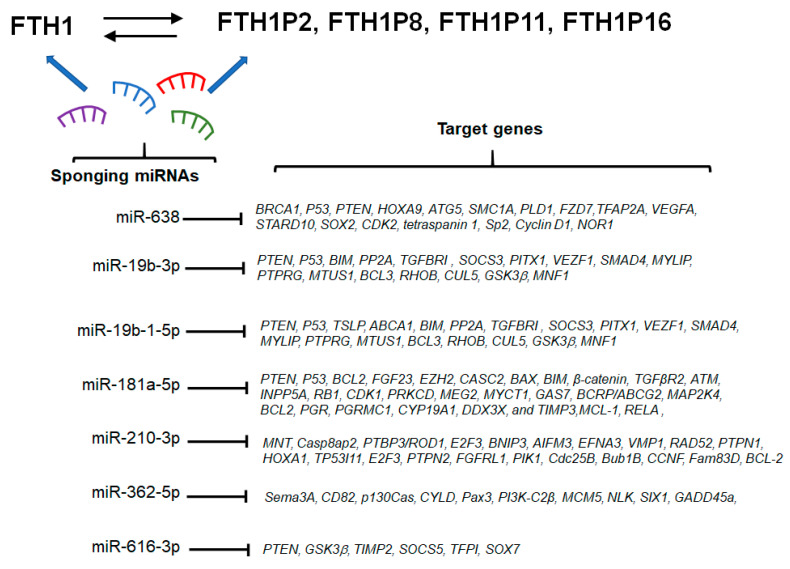
Potential networks involving *FTH1* gene/pseudogenes and known oncogenes or tumor suppressor genes. Abbreviations used: BRCA1, BRCA1 DNA repair associated; P53, Tumor protein p53; PTEN, Phosphatase and tensin homolog; HOXA9, Homeobox A9; ATG5, Autophagy related 5; SMC1A, Structural maintenance of chromosomes 1A; PLD1, Phospholipase D1; FZD7, Frizzled class receptor 7; TFAP2A, Transcription factor AP-2 alpha; VEGFA, Vascular endothelial growth factor A; STARD10, StAR related lipid transfer domain containing 10; SOX2, SRY-box transcription factor 2; CDK2, Cyclin dependent kinase 2; TSPAN1, Tetraspanin 1; Sp2, Sp2 transcription factor; CCND1, Cyclin D1; NOR1, Neuron-derived orphan receptor 1; BIM, Bcl-2-like protein 11; PP2A, Protein phosphatase 2 phosphatase activator; TGFBR1, transforming growth factor beta receptor 1; SOCS3, Suppressor of cytokine signaling 3; PITX1, Paired like homeodomain 1; VEZF1, Vascular endothelial zinc finger 1; SMAD4, SMAD family member 4; MYLIP, Myosin regulatory light chain interacting protein; PTPRG, Protein tyrosine phosphatase receptor type G; MTUS1, Microtubule associated scaffold protein 1; BCL3, BCL3 transcription coactivator; RHOB, Ras homolog family member B; CUL5, Cullin 5; GSK3β, Glycogen synthase kinase 3 beta; MNF1, Ubiquinol-cytochrome c reductase complex assembly factor 2; TSLP, Thymic stromal lymphopoietin; ABCA1, ATP binding cassette subfamily A member 1; BCL2, BCL2 apoptosis regulator; FGF23, Fibroblast growth factor 23; EZH2, Enhancer of zeste 2 polycomb repressive complex 2 subunit; CASC2, Cancer susceptibility 2; BAX, BCL2 associated X, apoptosis regulator; CTNNB1, β-catenin; TGFβR2, Transforming growth factor beta receptor 2; ATM, ATM serine/threonine kinase; INPP5A, Inositol polyphosphate-5-phosphatase A; RB1, RB transcriptional corepressor 1; CDK1, Cyclin dependent kinase 1; PRKCD, Protein kinase C delta; MEG2, Protein tyrosine phosphatase non-receptor type 9; MYCT1, MYC target 1; GAS7, growth arrest specific 7; BCRP/ABCG2, ATP binding cassette subfamily G member 2; MAP2K4, Mitogen-activated protein kinase kinase 4; PGR, Progesterone receptor; PGRMC1, Progesterone receptor membrane component 1; CYP19A1, Cytochrome P450 family 19 subfamily A member 1; DDX3X, DEAD-box helicase 3 X-linked; TIMP3, TIMP metallopeptidase inhibitor 3; MCL-1, MCL1 apoptosis regulator, BCL2 family member; RELA, RELA proto-oncogene NF-kB subunit; MNT,  MAX network transcriptional repressor; Casp8ap2, Caspase 8 associated protein 2; PTBP3/ROD1, Polypyrimidine tract binding protein 3; E2F3, E2F transcription factor 3; BNIP3, BCL2 interacting protein 3; AIFM3, Apoptosis inducing factor mitochondria associated 3; EFNA3, Ephrin A3; VMP1, Vacuole membrane protein 1; RAD52, RAD52 homolog DNA repair protein; PTPN1, Protein tyrosine phosphatase non-receptor type 1; HOXA1, Homeobox A1; TP53I11, Tumor protein p53 inducible protein 11; PTPN2, Protein tyrosine phosphatase non-receptor type 2; FGFRL1, Fibroblast growth factor receptor like 1; PIK1, PRKCA-binding protein; Cdc25B, Cell division cycle 25B; Bub1B, BUB1 mitotic checkpoint serine/threonine kinase B; CCNF, Cyclin F; Fam83D, Family with sequence similarity 83 member D; Sema3A, Semaphorin 3A; CD82, CD82 molecule; p130Cas, BCAR1 scaffold protein, Cas family member; CYLD, CYLD lysine 63 deubiquitinase; Pax3, Paired box 3; PI3K-C2β, phosphatidylinositol-4-phosphate 3-kinase catalytic subunit type 2 beta; MCM5,  Minichromosome maintenance complex component 5; NLK, nemo like kinase; SIX1, SIX homeobox 1; Gadd45a, Growth arrest and DNA damage inducible alpha; TIPM2, TIMP metallopeptidase inhibitor 2; SOCS5, Suppressor of cytokine signaling 5; TFPI, Tissue factor pathway inhibitor; SOX7, SRY-box transcription factor 7.

Another field that needs further investigation is the link between the *FTH1*/pseudogenes networks, the miRNAs involved in these networks, and other tumor genes. An example of such possible connections is depicted in Figure 4, which summarizes the possible connection between the *FTH1*/pseudogenes networks that have so far been experimentally demonstrated and oncogenes/tumor suppressor genes, based on common miRNA targets reported in the literature.

## Figures and Tables

**Table 1:** Features of *FTH1* pseudogenes.

Name	NCBI Reference	Location	Length (bp)	Evidence	Type	Putative Protein% Identity FTH1	Reference
*FTH1P1*	NG_004768.3	1p34.3	1096	INFERRED	PROCESSED	71aa (91.23%)	[57,58]
*FTH1P2*	NG_007330.2	1q42.13	663	VALIDATED	PROCESSED	113aa (94.69%)	[56,57,59,60]
*FTH1P3*	NR_002201.1	2p23.3	954	VALIDATED	PROCESSED	103aa (91.78%)	[57]
*FTH1P4*	NG_004769.6	3q21.3	1112	VALIDATED	PROCESSED	183aa (91.8%)	[57,61]
*FTH1P5*	NG_005639.3	6p12.3	1113	INFERRED	PROCESSED	79aa (92.41%)	[57]
*FTH1P6*	NG_043893.1	2p16.2	336	INFERRED	PROCESSED	50aa (0%)	Direct submission
*FTH1P7*	NG_007331.3	13q12.12	1122	INFERRED	PROCESSED	79aa (94.94%)	[57]
*FTH1P8*	NG_007336.2	Xq28	1112	VALIDATED	PROCESSED	87aa (95.12%)	[56,57,59]
*FTH1P9*	NG_043625.1	5q14.2	555	INFERRED	PROCESSED	45aa (0%)	Direct submission
*FTH1P10*	NG_003028.3	5p15.1	1119	VALIDATED	PROCESSED	253aa (95.78%)	[57,61]
*FTH1P11*	NG_007332.3	8q21.13	1108	VALIDATED	PROCESSED	113aa (96.46%)	[56,57,59,60,61]
*FTH1P12*	NG_007333.2	9p22.3	1115	VALIDATED	PROCESSED	152aa (91.16%)	[57]
*FTH1P13*	NG_007334.1	14q23.3	877	VALIDATED	PROCESSED	83aa (79.52%)	[61,62]
*FTH1P15*	NG_007335.3	6p12.1	655	INFERRED	PROCESSED	95aa (69.14%)	[58]
*FTH1P16*	NG_007337.2	11q14.1	1118	VALIDATED	PROCESSED	183aa (95.63%)	[56,57,59,63,64]
*FTH1P20*	NG_023542.2	2q31.3	1061	VALIDATED	PROCESSED	49aa (0%)	[61]
*FTH1P21*	NG_001121.4	4q32.1	1115	INFERRED	PROCESSED	108aa (0%)	[65,66,67]
*FTH1P23*	NG_025708.2	3p13	952	VALIDATED	PROCESSED	87aa (91.03%)	[61,65,66,67]
*FTH1P24*	NG_022082.2	4q31.1	747	INFERRED	PROCESSED	47aa (79.31%)	Direct submission
*FTH1P25*	NG_022745.1	1q25.3	671	INFERRED	PROCESSED	77aa (57.75%)	Direct submission
*FTH1P26*	NG_028813.1	6q23.2	714	INFERRED	PROCESSED	61aa (53.57%)	Direct submission
